# Are school difficulties an early sign for mental disorder diagnosis and suicide prevention? A comparative study of individuals who died by suicide and control group

**DOI:** 10.1186/s13034-019-0308-x

**Published:** 2020-01-14

**Authors:** Fabienne Ligier, Charles-Edouard Giguère, Charles-Edouard Notredame, Alain Lesage, Johanne Renaud, Monique Séguin

**Affiliations:** 1 McGill Group on Suicide Studies, Douglas Hospital, 6875, Boulevard LaSalle, Montreal, QC H4H 1R3 Canada; 2 Québec Network on Suicide Research, Mood Disorders and Related Disorders (RQSHA), Montreal, QC Canada; 3 EA 4360 APEMAC, Faculty of Medicine, Université de Lorraine, 54500 Vandoeuvre-lès-Nancy, France; 4 Centre Psychothérapique de Nancy, PUPEA, rue du Dr Archambault, 54520 Laxou, France; 5 Banque Signature, Research Center, Institut Universitaire en Santé Mentale de Montréal, 7401 Rue Hochelaga, Unit 218, Montreal, QC H1N 3M5 Canada; 6 Centre Hospitalier Régional Universitaire de Lille, 2 Avenue Oscar Lambret, 59037 Lille Cedex, France; 7 Department of Psychiatry, Université de Montréal, Montreal, QC Canada; 8 Institut Universitaire en Santé Mentale de Montréal, 7401 Rue Hochelaga, Unit 218, Montreal, QC H1N 3M5 Canada; 9 Manulife Centre for breackthroughs in Teen Depression and Suicide Prevention, Douglas Institute, 7070, Boulevard Champlain, Montreal, QC H4H 1R3 Canada; 10 Department of Psychoeducation and Psychology, Université du Québec en Outaouais, 283 Boulevard Alexandre-Taché, Gatineau, QC J8X 3X7 Canada; 11 Centre intégré de santé et service social de l’Outaouais (CISSSO), Outaouais, Canada; 12 Département Pédopsychiatrie, CHRU Nancy, Rue du Morvan, 54500 Vandoeuvre-lès-Nancy, France

**Keywords:** School difficulties, Mental disorders, Suicide, Child and adolescent

## Abstract

**Background:**

Suicide is the third leading cause of death worldwide among youth aged 10- to 19, and mental disorders are often associated in the etiology of suicidal behavior. Mental disorders are often under-diagnosed and under-treated in young people, a situation likely to increase the severity of the disorder and suicide risk. Presence of school difficulties may, in some cases, be a consequence of mental disorder, and theses difficulties are observable. Therefore, early detection and early intervention of school difficulties may alleviate the development of mental disorders and suicide vulnerability. The aim of this study is to understand the link between school difficulties and suicide risk.

**Methods:**

We used the data bank gathered by the McGill Group on Suicide Studies over the past two decades through interviews with the relatives of individuals who died by suicide and with individuals from the community as a control group. We included data on common sociodemographic characteristics, life events and mental health characteristics identified before age 18, among individuals who died before the age of 35 or were interviewed before the age of 35. We identified 200 individuals who died by suicide and 97 living controls. We compared groups according to gender and characteristics.

**Results:**

Within the total sample, 74% were male, 13% had met with academic failure, 18% had engaged in inappropriate behavior at school, and 18% presented combined school difficulties. Combined school difficulties (academic failure and inappropriate behavior) for both sexes and academic failure alone for males were associated with higher suicide risk before the age of 35. School difficulties generally began in early childhood and were linked to mental disorders/difficulties and substance abuse before age 18.

**Conclusions:**

This study underlines the importance for parents, teachers, and educators to identify children with school difficulties—academic failure and behavioral difficulties at school—as early as possible in order to be able to propose adapted interventions. Early identification and proper diagnosis may prevent chronicity of some disorders, accumulation of adverse events, and even suicide.

## Background

Suicide is the third leading cause of mortality worldwide among youth aged 10- to 19 [1]. Its etiology is characterized by multiple factors, both distal and proximal [2]. Distal or predisposing factors, which include heredity, early-life adversity, and personality traits, are involved in the emergence of impulse control difficulties, high introversion, conduct disorder, poor problem-solving capacity, and traumatic brain injury [3]. Most of these risk factors may appear early and may be observed only in the family environment and could go undetected until the child goes to school. Presence of school difficulties, which may be in some cases a consequence of a mental disorder [4], are observable and can be the first occasion for early detection and treatment [5].

As for proximal or precipitating factors, they include not only negative life events, such as death in the family, ongoing school difficulties, harassment, and financial difficulties, but also mental disorders, such as depression, substance abuse, and personality disorders, not to mention hopelessness, loneliness, perceived burdenson and thwarted belonging [2, 6]. Though mental disorders, especially depression and substance abuse, have been found to be a factor in 70% to 90% of suicides [7, 8], it has also been reported that 25.3% of individuals who died by suicide had no contact with psychiatric healthcare in the year prior to death [9]. Moreover, mental disorder has been found to be under-treated in 20% of children and adolescents affected [1, 10, 11]. One reason for this is the under-detection of symptoms, which can result in clinical misdiagnosis and poor care coordination [1, 12–14]. In addition, the fear of stigmatization has been pointed to as a key factor in the under-utilization of psychiatric services by young people and their parents [15]. Under-utilization, under-diagnosis and under-treatment translate into a lost opportunity for children and adolescents with a mental disorder. Indeed, when a psychiatric condition, such as anxiety or depression, goes undiagnosed and untreated, it has a higher risk of recurring and of doing so more severely than do treated disorders [16]. What’s more, an undiagnosed mental disorder may have not only short-term consequences, such as the adoption of risky behaviors, dropping out of school, and engaging in delinquency, but also longer-term consequences, such as alcohol abuse and impulsivity-violence [1]. All of these factors contribute to increase suicide risk.

Early intervention is key to prevent such adverse consequences and suicide. Early intervention, however, requires early diagnosis, which in turn requires early symptom detection. Symptoms and other telltale signs are more easily detected when objective: presence of school difficulties is one such sign. Indeed, according to the literature, school difficulties are linked to mental health vulnerability and suicidal behaviors [4, 5, 17–21]. A review of the literature on children’s mental health and “school performance”, “academic functioning”, “school attendance”, underlined the early manifestations of every mental health disorder of childhood, as identifiable behaviors affecting and affected by school performance” [5]. Chau et al. [17] found, among French teenagers, an association between poor psychological health and school absenteeism, after adjustment on gender, age, school-level and socioeconomic factors. Thompson et al. [4] showed that school difficulties in a population of 9- to 12-year-olds (US) are correlated with substance use, aggression, depression and suicidal behaviors very early on in life. In this study, presence of school difficulties was based on several criteria, as absence rate or grade points or credits earned. [4, 22]. Among teenagers with depression in New-Zeland, Fergusson et al., identified that school truancy and suspension were significantly associated with risk of suicide [20]. Among French teenagers, Chau et al. [18] reported that having to repeat a school year, increased risk for suicidal ideation in the last 12 months of interview, by a multiple of 1.51 (1.00–2.31 95% CI), and lifetime suicide attempt, by a multiple of 1.92 (1.21–3.04 95% CI). Walsh and Eggert [23], for their part, found risk for suicidal ideation and suicide attempts to be higher in a population of 14- to 21-year-olds with school difficulties than in the general samples US teens. These authors operationalized school difficulties with the criteria described by Herting [22]. In addition, Ligier et al. [21] observed that adolescent suicide attempters (French teenagers) with academic failure were at higher risk of recurrence during the 10 years following a first suicide attempt. Finally, in their study of suicide trajectories (Canadians), Séguin et al. [24] noted an association between academic difficulties and suicide. Depending on the study, school difficulties are operationalized differently and include different variables, such as academic failure, inappropriate behavior at school or absenteeism. These manifestations are not only indirect predictors of current or future mental health difficulties, but also, as highlighted in several studies, may as well be associated with past family difficulties and early-life adversity [19, 25, 26].

However, in most of the studies published to date, the different types of school difficulties have been assembled into one large variable. Consequently, it is difficult to ascertain the role and repercussions of each type of difficulty. Furthermore, past studies have generally focused on youth who had suicidal ideas or those who made a suicide attempt. In order to further the knowledge, we undertook a study to examine presence of school difficulties (academic failure and inappropriate behavior) and its co-occurrence on suicide before age 35. In this study, academic failure refers to learning difficulties—low grades and having to repeat a school year—, and inappropriate behavior at school is refered to difficulties with peers in a school setting, such as aggressive or provocative behaviors. Our purpose was to further our understanding in regards to the link between school difficulties and suicide risk and to determine whether early identification of this specific and easily detectable difficulties associated to suicide vulnerability might serve as early detection and prevention efforts.

## Methods

We used a retrospective comparative design in this study to compare individuals who died by suicide and living individuals as a control group.

### Participants and recruitment

Thanks to an ongoing partnership with the Office of the Quebec Coroner (OQC), a researcher at the McGill Group on Suicide Studies has been documenting for the past two decades the life trajectories of individuals who died by suicide by interviewing their bereaved family members. Following a suicide, the OQC sends the family an introductory letter explaining the research project, and family members are asked to call the research team. A trained mental health clinician then further explains the study and the interview procedure to the family. If the family members agree to participate in the study, an appointment is set for a first face-to-face interview. Generally speaking, the interviews take place within 3 to 4 months after the suicide. Two interviews, each of approximately 3 h long, are conducted by the same clinician. Approximately 75% of the family members contacted by the OQC agree to participate in the data collection.

A second group of individuals were interviewed in the course of various previous studies for comparison purposes [14]. Most were recruited via snowball sampling among the general population and were interviewed using the same measures as for the first group. All participants signed a consent form. The research was approved by the Research Ethics Boards of the Douglas Mental Health Institute (Montreal) and of the Université du Québec en Outaouais (for further details on the methods and confidentiality policy, see Séguin et al. [24]).

Life trajectories of more than 700 individuals who died by suicide and individuals assigned to control groups, ranging between 14 and 84 years of age, have been documented over the past two decades.

In order to limit memory bias, we selected from this data base individuals who died under 35 years of age and individuals in comparaison groups who were under 35 years of age at the time of the interview.

The study sample is composed of 200 individuals who died by suicide and 97 individuals as controls.

### Research instruments

Information on common sociodemographic characteristics, life events and mental health characteristics was collected from respondents during the face-to face interviews.

### Interview to determine post-mortem diagnosis

The psychological autopsy method was used to investigate individuals who died by suicide [27, 28]. During the interview, a researcher administered semi-structured questionnaires and the Structured Clinical Interview for DSM-IV for both Axis I and Axis II disorders (SCID I and II) [29, 30] to an informant who had known the deceased well, and to the control participants themselves. The procedure for the suicide group involves an interview with family members or close relatives, which was described previously in greater detail in Kim et al. [31] and in Dumais et al. [32]. As for the control group the interviews were conducted directly with the participant. For both groups, hospital records were examined to corroborate the informant’s report. A case vignette was then created on the basis of this information and submitted to a panel of experts for them to determine a post-mortem diagnosis or post interview diagnosis by consensus. A series of studies over the past decade have established agreement between DSM diagnoses based on informant report and those based on medical charts [33] and have shown the psychological autopsy method to be reliable [28, 34, 35]. However, according to research, the SCID-I does not identify childhood onset disorders like autism spectrum disorder, ADHD or oppositional disorders very well [29].

### Interview to retrace the life trajectory

The life trajectory interview was developped as a life history calendar research [36, 37]. The life calendar is used as an aid to accurately retrace the major events and significant experiences in an individual’s life. The calendar explores a number of clearly described variables from all spheres of life. Following the interview, a clinical case history (case vignette) and a life calendar are created, in accordance with the narrative research method [38]. The life calendar makes it possible to pinpoint the occurrence of specific events, both positive and negative. The frequency, duration and severity of each event is recorded and the event is classified in one of the following life spheres: early adversity (e.g., abuse, neglect, presence of violence); academic life (e.g., path, interruptions, success, failure); professional life (e.g., unemployment, stress at work, promotions); social life (e.g., presence or absence of social support, friends, colleagues); and interpersonal difficulties (e.g., difficulties associated with mental health problems, suicide attempts, illness). This methodology has been presented in depth elsewhere [8]. For this study, variables associated with presence of school difficulties and mental health problems were considered.

### Data analysis

Data analysis was performed on R version 3.3 [39]. We used the *lavaan* package [40] for Structural Equation Modeling (SEM).

The following childhood risk factors (< 18 years of age) were coded during the interview and correspond with the variables identified below:

- academic failure (i.e. learning difficulties – low grades and having to repeat a school year);
- inappropriate behavior at school (i.e., difficulties associated with social life at school and behavior difficulties in school setting, such as aggressive or provocative behavior with peers or teachers);
- combined school difficulties (academic failure + inappropriate behavior at school);
- mental health difficuties and disorders, i.e. mental health problems, including DSM-IV Axis 1 and 2 mental health disorders, identified through the SCID I (Axis 1) and II (Axis 2) + clinical mental health difficulties without complete diagnosis in the DSM-IV;
- substance abuse, as identified through the SCID-I.

First, we calculated the distribution (number, percentage, mean, standard deviation) of the characteristics considered in the study for the two groups respectively: individuals who died by suicide (n = 200) and individuals in the control group (n = 97) before age 35. We then used Chi-square to compare the distributions both between the groups and within the groups by sex.

Secondly, we employed SEM to assess the correlation between the characteristics considered for the suicide group. This method uses confirmatory factor analysis of covariance matrices to examine structural relationships. We described the pattern of characteristics between the gender. Third, we used Chi square again to compare both groups in terms of age at onset of school difficulties and other risk factors considered. Age at onset was dichotomized as follows: under 12, and 12 and over. Twelve corresponds to the age at which children enter the seventh grade (high school) in Quebec and may be the beginning of puberty [41, 42]. The younger age group—under 12—was analysed in order to explore the presence of early onset of school difficulties and mental health problems [43, 44]. The significance threshold was set at 0.05 for all the analyses.

## Results

In the total sample (N = 297), 74% were male, 13% had met with academic failure, 18% had engaged in inappropriate behavior at school, and 18% presented combined school difficulties. The sample was composed of 200 individuals who died by suicide and 97 individuals who are in the control group, who were all under the age of 35. For the suicide group, mean age of death was 22 years (SD = 5.5). For the control group, mean age at interview was 22.6 (SD = 4.7).

### Characteristics by group are presented in Table 1

Males made up a larger proportion of the suicide group. All differences between individuals who died by suicide and those who were in a control group were statistically significant (p < 0.05), except for inappropriate behavior at school (p = 0.66). In addition, among individuals who died by suicide, substance abuse was present among 55% of males vs. 37% of females (p = 0.04). There was no other significant difference between males and females.

**Table 1:** Characteristics of suicides and controls (N = 297)

Characteristics	Individuals who died by suicide n = 200 *% (n)*	Individuals in control group n = 97 *% (n)*	p*
Male gender	81 (162)	59 (57)	< *0.0001*
Academic failure	16 (32)	6 (6)	*0.02*
Inappropriate behavior at school	18 (35)	20 (19)	0.66
Combined school difficulties	23 (45)	9 (9)	*0.006*
Mental disorders/difficulties	60 (120)	35 (34)	< *0.0001*
Substance abuse	52 (103)	19 (18)	< *0.0001*

* Bivariate analysis with Chi-square test to compare suicides and controls, significant at *p* < 0.05 (in italics)

**Fig. 1:**
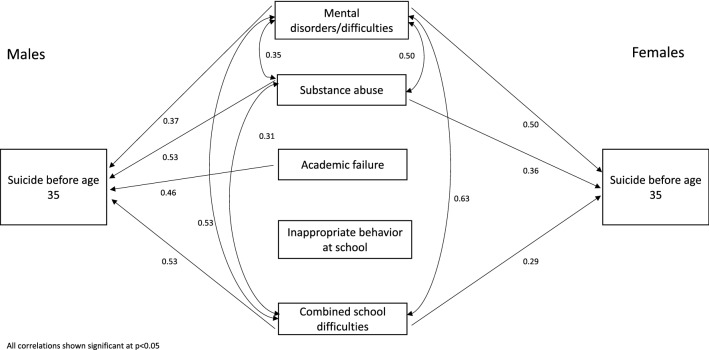
Structural equation model of correlation between school difficulties, mental health characteristics and suicide, by sex

Figure 1 presents the structural equation model of the correlations between the characteristics considered for individuals who died by suicide before age 35, by gender (n = 200). Only the correlations that proved statistically significant are represented. For males (n = 162), who died by suicide before the age of 35 years, correlated with academic failure (0.46, p = 0.001), combined school difficulties (0.53, p = 0.0001), mental disorders/difficulties (0.37, p = 0.0001), and substance abuse (0.53, p = 0.0001). For females (n = 38), who died by suicide before the age of 35 years, correlated with combined school difficulties (0.29, p = 0.01), mental disorders/difficulties (0.50, p < 0.0001), and substance abuse (0.36, p = 0.04). Correlations emerged also between the variables considered. Mental disorders/difficulties correlated with substance abuse and with combined school difficulties for both sex. Substance abuse correlated with combined school difficulties for males only (see Fig. 1).

Regarding age at onset of school difficulties (see Table 2), academic failure, inappropriate behavior at school, and combined school difficulties appeared in majority before 12 (from 47 to 91%). Inter-group differences were not statistically significant.

**Table 2:** Inter-group comparison of onset of school difficulties before age 12 (N = 146)

Characteristics	Individuals who died by suicide with school difficulties *n (percentage)*	Individuals in control group with school difficulties *n (percentage)*	p*
Academic failure, onset before age 12	*n *= *32* 20 (63)	*n *= *6* 5 (83)	0.32
Inappropriate behavior at school, onset before age 12	*n *= *35* 23 (66)	*n *= *19* 9 (47)	0.19
Combined school difficulties, onset before age 12	*n *= *45* 41 (91)	*n *= *9* 8 (89)	0.83

* Bivariate analysis with Chi-square test to compare suicides and controls, significant at p < 0.05

Finally, for the sample with school difficulties before age 12 including both groups (n = 146), onset of academic failure before age 12 was associated with higher risk for mental disorders/difficulties (relative risk = 10.9 [1.1–105.8], p = 0.02).

## Discussion

This study confirms that combined school difficulties for both gender, and academic failure for males are associated with higher risk for suicide before the age of 35. Moreover, as reported in other studies, school difficulties generally begin in early childhood and are correlated with the presence of mental health difficulties or disorders and substance abuse before 18 years of age. These findings suggest that early identification of school difficulties and mental disorders/difficulties in childhood might be an important avenue to pursue in suicide prevention.

School difficulties have a major impact on the psychological development of children. When children or adolescents fail at school—and, as we observed from the data in this study, school difficulties generally begin in early childhood—their entire identity during the development process may be affected by a lowered sense of self-esteem; it may also impair their capacity to seek help, increase their vulnerability to anxiety, and may have long-term consequences [13]. Link between academic failure and suicide was observed in our study regardless of the origin of academic failure. Learning disabilities, which are associated to academic failure, affect from 2 to 10% of 10-year-old students. Learning disabilities include difficulties acquiring, organizing, understanding, and using verbal and non-verbal information [43, 45]. Pan et al. demonstrated a direct link between academic failure and risk for major depression among adolescents [46]. Given that depression in children and adolescents may sometimes be expressed in the form of irritability and even aggression, some inappropriate behavior at school may be a symptom of this disorder. Inappropriate behavior at school may also result from anxiety and impulse control, which median age at onset was found to be 11 ears of age-at-onset in the Kessler study of DSM-IV disorders [44]. Even if aggressive behaviors, are common at age 3, as described by Tremblay [47], if these behaviors become chronic, they may impact the child’s or adolescent’s future. In a longitudinal study of 10 year old children in the province of Quebec, (2001 to 2008), 18.8% of children have been diagnosed with mental disorder and proportionally more boys than girls presented externalizing symptoms, such as ADHD [43]. Specifically, ADHD affects from 9 to 19% of children age 10 in Quebec, but it was underdiagnosed 30 years ago, that is, in the childhood period of the participants we studied [43]. If we consider the sample in this study we could make the hypothesis that some of them, may have had a non-diagnosed attention deficit hyperactivity disorder (ADHD). As indicated earlier, the SCID-I was not designed to screen for early-childhood-onset disorders such as ADHD and oppositional disorders [29]. We may suppose that children with inappropriate behavior at school tend to be more impulsive and to have more relational difficulties—two symptoms associated with ADHD. This disorder may also be associated with academic failure and/or learning disabilities. When ADHD goes undiagnosed, there may be a higher risk for substance abuse, a factor at play in half of all suicide deaths [8, 48–50].

In our study, only males were at higher risk for suicide when they met with academic failure alone. Generally speaking, females are more likely than males to attempt suicide, but females with externalizing symptoms, such as aggressive behavior or a conduct or oppositional disorder are at higher risk for suicide owing to their impulsivity [51]. This may explain why, for them, combined school difficulties (i.e., inappropriate behavior at school and academic failure) and not just academic failure constitute a risk factor for suicide before age 35. However, we have to keep in mind that males are over represented in the sample of individuals who died by suicide, as in suicide in general.

From the study results, it seems possible to decrease suicidal behaviors through early identification of mental disorders/difficulties and the earliest possible intervention, given that half of all lifetime cases of mental disorder start before age 14 [44]. Another reason strongly supporting early diagnosis is that it is easier to involve children and adolescents in treatment when parental support is available, than later on in young adulthood when such support might be harder to come by. Moreover, in cases of inadequate family background, school professionals might help children and adolescents to access treatment, directly in the school environment [5, 25, 26]. Because children and adolescents do not seek professional help for fear of stigmatization and lack of confidentiality [15, 52], that underlines the importance to communicate on mental health services to defuse prejudices.

Our study has various limitations stemming from the retrospective method used, uppermost among these are memory biases. The life calendar interview, is similar to a clinical interview and was used for collecting data in order to minimize memory bias [36, 37]. Even if data have not been collected directly for individuals who died by suicide, a series of studies over the past decade have established agreement between DSM diagnoses based on informant report and those based on medical charts, or between proxi-based data in suicide research [27, 33]. In this study, we considered the association between the variables, in a cross-sectional manner, which does not allow examining the evolution of these characteristics over time. Finally, as is often the case in this type of study, controls were not exactly representative of the general population, as they were generally recruited among friends and neighbours of suicides, that share environmental determinants of mental disorders, as evidenced in Lesage et al.’s youth suicide case–control study [53]. This may have introduced a Berkson’ bias in the sampling, which might explain the high rate of mental disorder among our controls [54]. There are more females in the control group: an over-diagnosis of inappropriate behavioral at school may result in suicide group as an over-representation of academic failure. To limit this bias, we used SEM in multivariate analysis by gender only for individuals who died by suicide.

This study also has two principal strong points. First, it is original in that this type of research has never been conducted retrospectively among individuals who died by suicide. Second, the school difficulties examined are easy to identify in real life by teachers, family members and all adults who work with children and adolescents. Third, we use the threshold of 35 to increase the relevance of the results for contemporary health planning and policy making.

Consequently, if school difficulties prove to be an early sign of mental illness or, at the very least, of vulnerability, then they might serve as an objective and easily detectable flag for early intervention in order to improve the immediate and future welfare of children and adolescents. In this regard, the Quebec Ministry of Education recently recommended a series of measures for the early identification and support of children at risk for or presenting school difficulties [55, 56]. Recognizing the presence of school difficulties might make it easier to diagnose the five most common childhood mental disorders, as these disorders are associated with such difficulties. The five disorders in question are ADHD, mood disorder (anxiety/depression), substance abuse, oppositional defiant disorder, and suicidal behavior. Once a diagnosis is established, children and adolescents can then receive adapted treatment [57]. For Heckman and Masterov [58], academic failure carries social and economic costs. In order to decrease school dropout and academic failure and to increase rates of high school graduation and college attendance, these authors recommend pre-school interventions targeted at children in disadvantaged environments. Universal prevention programs, such as those to prevent conduct disorder, have been developed in order to improve prosocial behavior and/or decrease antisocial behavior [59]. These are provided in schools to 6-year-olds in Canada [60] and to 5- to 9-year-olds in the United States [61]. Finally, improving the quality of life of young students and reducing their suicide risk through early intervention can translate into considerable cost savings for the public sector over the long run, as argued by Knapp et al. in promoting School-based social and emotional learning programs [62]. Along similar lines, Heckman and Masterov [58] have advocated investing in early childhood education as a cost-effective strategy to stimulate economic growth.

Besides prevention and detection of school difficulties, some researchers have proposed screening children at school entry for risk factors for developing mental health problems, but the strategy is a very expensive one [63]. As a low cost alternative, this screening could be done using a computerized instrument such as the Dominique Interactive, which allows screening for mental disorders reliably through game-like testing that appeals to children [64]. The Dominique Interactive, specifically, is available in two versions: one for children 6 to 11 and the other for adolescents 12 to 16 [65]. Studies to date have demonstrated its validity, reliability, and psychometric properties, but it has yet to be the subject of an implementation study.

At last, even if early diagnosis for early care is important, professionals have to be cautious of the risk of over-diagnosis: not all children with school difficulties have mental health disorders. Every case needs a specific evaluation without stigmatization or judgment.

## Conclusions

This study underlines the importance for parents, teachers, and educators to recognize children with school difficulties—academic failure and inappropriate behavior at school—as early as possible given the link between these manifestations and higher risk for mental disorders/difficulties, substance abuse, and suicide before age 35 and the difficulty for children and adolescent for seeking help when needed. Once school difficulties are acknowledged, it is essential then to propose adapted interventions, including psychoeducation, mental health care, and remediation in the case of learning disabilities. Early identification and proper diagnosis may prevent some disorders from becoming chronic, adverse events from accumulating, and suicide from becoming the only option.

Finally, more research is needed on school and behavioral difficulties before we can determine whether or not these characteristics are direct risk factors for suicide.

## Data Availability

The datasets used and/or analyzed during the current study are available from the corresponding author on reasonable request.

## Abbreviations

ADHD: attention deficit hyperactivity disorder
OQC: Office of the Quebec Coroner
SCID: Structured Clinical Interview for DSM-IV
SEM: structural equation modeling
